# Supplementary material for: Multi‐dataset identification of innovative feature genes and molecular mechanisms in keratoconus
Source: J Cell Mol Med. 2024 Sep 19;28(18):e70079. doi: 10.1111/jcmm.70079 (PMC11412914; doi:10.1111/jcmm.70079)
Supplement: Supplementary file 2 — Material S2. [file JCMM-28-e70079-s002.zip › 7.GSEA analysis/5.RNF217-AS1/KEGG_RIG_I_LIKE_RECEPTOR_SIGNALING_PATHWAY.html]

Details for gene set KEGG\_RIG\_I\_LIKE\_RECEPTOR\_SIGNALING\_PATHWAY[GSEA]

|  || Dataset | RNF217-AS1.group.cls#h\_versus\_l |
| Phenotype | group.cls#h\_versus\_l |
| Upregulated in class | h |
| GeneSet | KEGG\_RIG\_I\_LIKE\_RECEPTOR\_SIGNALING\_PATHWAY |
| Enrichment Score (ES) | 0.18776344 |
| Normalized Enrichment Score (NES) | 0.7058097 |
| Nominal p-value | 0.9085239 |
| FDR q-value | 0.9931915 |
| FWER p-Value | 1.0 |
Table: GSEA Results Summary

  

Fig 1: Enrichment plot: KEGG\_RIG\_I\_LIKE\_RECEPTOR\_SIGNALING\_PATHWAY      
 Profile of the Running ES Score & Positions of GeneSet Members on the Rank Ordered List

  

| SYMBOL | TITLE | RANK IN GENE LIST | RANK METRIC SCORE | RUNNING ES | CORE ENRICHMENT || 1 | IFNA14 | na | 927 | 0.451 | 0.0361 | Yes |
| 2 | IFNA4 | na | 1517 | 0.391 | 0.0737 | Yes |
| 3 | IL12A | na | 1615 | 0.381 | 0.1241 | Yes |
| 4 | IFNA16 | na | 1944 | 0.354 | 0.1640 | Yes |
| 5 | IFNA6 | na | 2619 | 0.310 | 0.1878 | Yes |
| 6 | IKBKB | na | 4368 | 0.238 | 0.1706 | No |
| 7 | TRAF3 | na | 7248 | 0.146 | 0.1079 | No |
| 8 | CASP10 | na | 8136 | 0.125 | 0.0997 | No |
| 9 | TRIM25 | na | 8875 | 0.108 | 0.0934 | No |
| 10 | MAPK12 | na | 9095 | 0.103 | 0.1015 | No |
| 11 | IFNA7 | na | 9097 | 0.103 | 0.1159 | No |
| 12 | DDX3X | na | 9293 | 0.099 | 0.1241 | No |
| 13 | CXCL8 | na | 10297 | 0.081 | 0.1065 | No |
| 14 | IFIH1 | na | 10636 | 0.076 | 0.1072 | No |
| 15 | IFNE | na | 10666 | 0.075 | 0.1169 | No |
| 16 | TRAF2 | na | 10890 | 0.072 | 0.1205 | No |
| 17 | MAPK13 | na | 10953 | 0.071 | 0.1285 | No |
| 18 | IFNA21 | na | 11545 | 0.062 | 0.1201 | No |
| 19 | AZI2 | na | 11694 | 0.060 | 0.1242 | No |
| 20 | MAPK11 | na | 12121 | 0.054 | 0.1194 | No |
| 21 | IFNA2 | na | 12465 | 0.049 | 0.1164 | No |
| 22 | IRF3 | na | 12763 | 0.045 | 0.1141 | No |
| 23 | IFNB1 | na | 12820 | 0.045 | 0.1187 | No |
| 24 | MAPK9 | na | 12895 | 0.044 | 0.1226 | No |
| 25 | TANK | na | 13551 | 0.036 | 0.1087 | No |
| 26 | TBKBP1 | na | 13573 | 0.035 | 0.1130 | No |
| 27 | TNF | na | 13583 | 0.035 | 0.1177 | No |
| 28 | SIKE1 | na | 13817 | 0.032 | 0.1155 | No |
| 29 | MAVS | na | 13994 | 0.031 | 0.1147 | No |
| 30 | MAPK14 | na | 14311 | 0.027 | 0.1093 | No |
| 31 | RIPK1 | na | 14579 | 0.024 | 0.1049 | No |
| 32 | MAPK10 | na | 15620 | 0.013 | 0.0767 | No |
| 33 | CHUK | na | 16613 | 0.003 | 0.0485 | No |
| 34 | MAPK8 | na | 16681 | 0.002 | 0.0468 | No |
| 35 | TRADD | na | 17611 | -0.007 | 0.0210 | No |
| 36 | IRF7 | na | 18108 | -0.012 | 0.0083 | No |
| 37 | TBK1 | na | 18209 | -0.013 | 0.0072 | No |
| 38 | NFKBIB | na | 18425 | -0.015 | 0.0031 | No |
| 39 | CASP8 | na | 18593 | -0.016 | 0.0006 | No |
| 40 | PIN1 | na | 18740 | -0.018 | -0.0012 | No |
| 41 | OTUD5 | na | 19480 | -0.025 | -0.0190 | No |
| 42 | FADD | na | 19613 | -0.026 | -0.0191 | No |
| 43 | TRAF6 | na | 20014 | -0.030 | -0.0265 | No |
| 44 | MAP3K7 | na | 20426 | -0.034 | -0.0335 | No |
| 45 | NFKB1 | na | 20531 | -0.035 | -0.0316 | No |
| 46 | IKBKG | na | 20686 | -0.037 | -0.0309 | No |
| 47 | RELA | na | 21550 | -0.046 | -0.0495 | No |
| 48 | ATG5 | na | 21857 | -0.049 | -0.0514 | No |
| 49 | DDX3Y | na | 22067 | -0.052 | -0.0503 | No |
| 50 | NLRX1 | na | 22173 | -0.053 | -0.0460 | No |
| 51 | CYLD | na | 24483 | -0.082 | -0.1011 | No |
| 52 | ATG12 | na | 24614 | -0.084 | -0.0931 | No |
| 53 | NFKBIA | na | 24662 | -0.085 | -0.0825 | No |
| 54 | MAP3K1 | na | 24706 | -0.086 | -0.0718 | No |
| 55 | IL12B | na | 24779 | -0.087 | -0.0617 | No |
| 56 | DHX58 | na | 26139 | -0.110 | -0.0857 | No |
| 57 | ISG15 | na | 29063 | -0.178 | -0.1452 | No |
| 58 | IFNA8 | na | 29826 | -0.201 | -0.1391 | No |
| 59 | IFNW1 | na | 30330 | -0.218 | -0.1233 | No |
| 60 | IKBKE | na | 30484 | -0.223 | -0.0966 | No |
| 61 | IFNK | na | 30871 | -0.237 | -0.0747 | No |
| 62 | IFNA10 | na | 32398 | -0.317 | -0.0746 | No |
| 63 | CXCL10 | na | 34045 | -0.491 | -0.0537 | No |
| 64 | RNF125 | na | 34199 | -0.526 | 0.0153 | No |
Table: GSEA details [plain text format]

  

Fig 2: KEGG\_RIG\_I\_LIKE\_RECEPTOR\_SIGNALING\_PATHWAY      
 Blue-Pink O' Gram in the Space of the Analyzed GeneSet

  

Fig 3: KEGG\_RIG\_I\_LIKE\_RECEPTOR\_SIGNALING\_PATHWAY: Random ES distribution      
 Gene set null distribution of ES for **KEGG\_RIG\_I\_LIKE\_RECEPTOR\_SIGNALING\_PATHWAY**
